# Fighting for the cures of rare genetic diseases: worthy ideas and promising results demand for strategic research management and for integration of competences and efforts

**DOI:** 10.1186/1750-1172-7-S2-A21

**Published:** 2012-11-22

**Authors:** Francesca Sofia

**Affiliations:** 1 Fondazione Telethon, Milan, Piazza Cavour 1, 20100, Italy

## 

The fight against genetic diseases, whose aim is the amelioration of the quality and prospect of life of the patients, poses enormous challenges due to several well-known issues such as the rarity of the diseases, the scarcity of financial resources and the lack of knowledge/interest from major stakeholders of the healthcare sector. To cope with this complex and multifaceted problem, in 1990 a patient association in Italy (UILDM) founded Telethon whose mission is to advance scientific research on muscular dystrophy and all other genetic diseases with the ultimate goal of paving the way for the cures. To this end, Telethon has set an articulated strategy whose pillars are: strategic funding of science, selection of the most promising and worthy research ideas, efficient management of funded projects and exploitation of research results. Indeed, in 22 years of activity, Telethon has invested about 354 million Euros, funding 2,375 projects on more than 450 genetic diseases. Notably, Telethon research spans all steps of the research path from the most basic and preliminary phases to the clinical translation, and is increasing our understanding of many genetic diseases, as demonstrated by a wealth of high impact publications. Moreover, clinical trials for different diseases are on-going or in the process to start.

This strategy has demonstrated itself as effective in advancing biomedical research; nonetheless, it is clear that the completion of the path to make therapies available to the patients requires resources and expertise that are beyond Telethon’s capacity and that typically reside within the competence of the pharmaceutical industry. This awareness has recently led Telethon to sign an agreement with a major pharmaceutical company that will allow gene therapies, performed at the San Raffaele-Telethon Institute for Gene Therapy, to enter the last phases of the developmental process for seven different genetic diseases.

The success of this agreement set the course of what is acclaimed as “collaborative model” in the field of rare diseases.

To date, there are several basic or pre-clinical research projects in Telethon’s portfolio that constitute a reservoir of ideas and knowledge on many different genetic diseases and deserve exploitation. In this perspective, partnerships with pharmaceutical industries and with other stakeholders will not only lead to the completion of the path for the most advanced research projects but also free-up resources to be invested in research activities at earlier stages of development, thus feeding a virtuous circle.

